# Knowledge, Practice, and Associated Factors of Insulin Self‐Administration in Patients With Diabetes at Dessie City Governmental Hospital Follow Up Clinic, Amhara Region, North East Ethiopia: Cross‐Sectional Study

**DOI:** 10.1002/hsr2.70631

**Published:** 2025-04-10

**Authors:** Wasihun Melaku Feleke, Caridad Sanchez Olis, Adem Hussien Endris, Seid Legesse Hassen, Yimer Seid Ali, Dessale Abate Beyene

**Affiliations:** ^1^ Department of Adult Health Nursing College of Health Science Wollo University Dessie Ethiopia; ^2^ Amhara Public Health Institute, Research Directorate Bahir Dar Ethiopia; ^3^ CDC, Amhara Regional Health Bureau Dessie Ethiopia; ^4^ Department of Pharmacy, Asrat Woldeyes Health Science Campus Debre Berhan University Debre Berhan Ethiopia

**Keywords:** diabetes mellitus, Ethiopia, knowledge, practice, self‐administration of insulin

## Abstract

**Background and Aims:**

Diabetes mellitus is an increasingly prevalent medical condition. The primary method of treating high blood sugar levels is subcutaneous insulin therapy. However, insufficient knowledge and poor practice related to insulin can negatively impact its effectiveness and adherence. Diabetic patients who are unaware of proper insulin self‐administration can experience severe complications. Hence, the aim of this study was to assess knowledge, practices, and associated factors related to the self‐administration of insulin among diabetic patients.

**Methods:**

An institutional cross‐sectional study used interviewer‐administered questionnaires from April 1, 2022, to June 30, 2022. Descriptive statistics were used to summarize sociodemographic data. Univariate and multivariate regression analyses were performed to measure the associations between the dependent and independent variables. A *p* value of less than 0.05 was generally considered statistically significant.

**Results:**

In this study, 69.3% of participants had good knowledge and 63.9% had good practice. In multiple logistic regressions, patients' occupation, type of diabetes, membership in the Ethiopian Diabetic Association, and the source of information about diabetes (from health professionals or media) were associated with knowledge of self‐administration of insulin. On the other hand, younger age (18–24 years) and duration of insulin therapy > 9 years were associated with good practice of self‐administration of insulin.

**Conclusion:**

The study found that patients had suboptimal knowledge and practice regarding insulin self‐administration. Patients must receive adequate education to address these knowledge gaps and improve insulin therapy outcomes.

**Abbreviations**
DM: diabetes mellitus
EDA: Ethiopian Diabetic Association
IDFA: International Diabetic Federation Association
T1DM: type 1 diabetes
T2DM: type 2 diabetes
WHO: World Health Organization

## 1 Introduction

Diabetes mellitus (DM) is a metabolic disorder characterized by chronic hyperglycemia with impaired carbohydrate, fat, and protein metabolism. It is caused by insufficient insulin production by the pancreas or because the body cells do not effectively use or respond to the insulin that is produced [1, 2]. Insulin plays a critical role in the control of DM, and multiple daily injections of insulin may be required to maintain normal blood glucose levels [3]. Insulin has been of paramount importance for the control of both type 1 diabetes (T1DM) and type 2 diabetes (T2DM) [4, 5]. To keep blood glucose levels under control, several doses of insulin had to be injected daily, which was essential for controlling and preventing complications of diabetes [6].

Insulin was one of the available antidiabetic drugs and also the most effective agent to reduce hyperglycemia when used in the correct dosage [7]. Patients with T1DM were treated with insulin injections in multiple doses or with continuous subcutaneous insulin infusions to control the load, as directed by the healthcare provider. The insulin injection technique was one of the most common areas where errors occurred. It required in‐depth knowledge and practice for patients to self‐administer insulin [8]. Injection of insulin was essential for the treatment of patients with T2DM and was also required by patients with T2DM for intermittent or continuous glucose control. The dosage of insulin should be consistent and the patient's injection technique should be reviewed regularly by the diabetes team [9].

The global prevalence of diabetes in 2019 was estimated to be 9.3% (463 million people), projected to increase to 10.2% (578 million) by 2030 and 10.9% (700 million) by 2045 [10]. DM has become one of the largest chronic health issues in Ethiopia, with more than 1.8 million diabetes patients and a national prevalence of 4.36% among the adult population [11]. In addition, the IDFA reported that Ethiopia ranked third among the top 10 countries in Africa, with 1.4 million DM cases and an estimated prevalence of 3.32% in 2012 [12].

Inadequate knowledge and practice related to insulin can likely affect its acceptance and adherence [13]. Insulin is an injectable drug, and therefore its users are more prone to misunderstandings than those using oral antidiabetic drugs [14]. It is essential to assess their knowledge and practice regarding self‐administration of insulin to identify and address any gaps. Self‐administration of insulin requires cognitive and psychomotor skills, and learning different procedures is crucial [15]. In Ethiopia, inadequate self‐management of diabetic patients, particularly patients' knowledge and practices regarding self‐administration of insulin, remains a significant problem facing healthcare providers and populations in all sectors. Inadequate self‐management impacts patient morbidity and mortality and increases medication and laboratory testing costs, as well as the time and effort required by healthcare providers. Adequate patient education at the beginning of treatment can go a long way toward reducing fears and misunderstandings because insulin therapies are complicated techniques that cannot be mastered simply by providing health education once or twice [16].

Insufficient knowledge about insulin can lead to errors in its use, which can lead to lipodystrophies, malabsorption, and reduced insulin effectiveness or inadequate treatment outcomes in patients [17]. Lack of awareness among diabetic patients regarding self‐administration of insulin can cause serious complications [19]. It is imperative to explore potential factors that affect insulin therapy to develop a strategy for optimizing it [20]. In Ethiopia, few studies address knowledge and practice toward self‐administration of insulin. Moreover, very few studies have examined all potential factors that influence knowledge and practice. Therefore, this study aims to assess knowledge, practices, and associated factors toward self‐administration of insulin among diabetic patients at Dessie Town Governmental Hospitals.

## 2 Methods and Materials

### 2.1 Study Setting

Dessie town was the capital of the South Wollo Zone in Amhara Regional State. It was located on the northeastern edge of the north‐central highlands of Ethiopia. There were two government hospitals in Dessie: the Dessie Referral Hospital (DRH) and the Boru Meda Hospital (BMH). Dessie Referral Hospital was established in 1962 during the reign of Emperor Halie Selassie, and there was only one diabetic outpatient clinic. Boru Meda Hospital was a primary hospital in the town of Dessie in the northeast of the country. It was founded in 1955 by the Sudanese Home Mission.

### 2.2 Study Design, Period, and Population

An institutional cross‐sectional study was carried out by using interviewer‐administered questionnaires to assess knowledge, attitude, and practice of insulin self‐administration among patients with DM conducted from April 01, 2022, to June 30, 2022. All patients treated for DM in the adult diabetes clinics at Dessie Town Governmental Hospital were considered the source population, and the study population was all adult patients treated for DM in the adult diabetes clinics at Dessie Town Governmental Hospital who met the eligibility criteria during the study period.

### 2.3 Eligibility Criteria

Inclusion criteria: (i) patients with confirmed T1DM or T2DM, (ii) DM patients taking insulin, (iii) age ≥ 18 years, (iv) ability to give informed consent, and willingness to participate in the study were included. Patients with cognitive impairment affecting participation in the study were excluded from the study.

### 2.4 Sample Size Determination and Sampling Procedures

To conduct our study, we used a single proportional formula [21] to calculate the sample size. We took the anticipated proportion of patients who self‐administer insulin, which is 54.4%, based on a previous study conducted at Hawassa Referral Hospital [22]. Our significance level (*α*) was set at 0.05, with a confidence interval (CI) of 95% and a margin of error (*d*) of 5%. To calculate the sample size, we used the following formula:

$$\begin{array}{l} n=\frac{\left({Z\frac{\alpha }{2})}^{2}\times p(1-P\right)}{d^{2}}\\ n=\frac{\left({1.96)}^{2}\times 0.54(1-0.54\right)}{{\left(0.05\right)}^{2}}=382 \end{array}$$

In this study, by adding a 10% nonresponse rate, the final calculated sample size was 421. The researchers used systematic random sampling, with proportionate allocation, to select study subjects from public health institutions in DRH and BMH.

### 2.5 Data Collection and Management

Data was collected using semi‐structured questionnaires adopted from various literature [6, 9, 14, 15, 23]. The questionnaire was divided into three sections. The first section aimed to gather the sociodemographic and clinical characteristics of the study participants. The second section included questions related to the knowledge of insulin self‐administration. The third section focused on the practice of insulin self‐administration in patients with DM at the Dessie City Governmental Hospital follow‐up clinic in the Amhara region of northeast Ethiopia.

### 2.6 Data Quality Assurance

Before conducting the main study, a pretest was conducted on 5% of the sample size outside the study area to ensure data quality. Based on the results of the pretest, the clarity and consistency of the data collection instrument were checked. The study director trained the data collectors, who were recruited by four clinical nurses, for half a day on the objectives of the survey and how to use the instrument to collect data from participants. To ensure consistency and completeness of the data, the study director reviewed all collected data daily to ensure data quality.

### 2.7 Data Analysis

The data were then entered into the Statistical Package for the Social Sciences (SPSS, IBM Corporation, Armonk, New York, the United States) version 25 for the final analysis. Descriptive statistics, including frequencies, percentages, means, and standard deviations, were utilized to analyze the sociodemographic variables. After checking the assumptions, multiple logistic regression analysis was performed to identify possible factors associated with knowledge and practice toward self‐administration of insulin. The univariate analysis was performed to obtain candidate variables for the multivariate regression models. In the univariate analysis, factors associated with knowledge and practice toward self‐administration of insulin with a *p* value of < 0.2 were considered candidates for a multivariate binary logistic regression model to identify strong factors associated with knowledge and practice toward self‐administration of insulin. A *p* value of < 0.05 was used to determine statistical significance.

### 2.8 Operational Definition

#### 2.8.1 Good Knowledge

Respondents were considered to have a “good knowledge” of insulin self‐administration if they answered at or above the mean score of the knowledge assessment questions.

#### 2.8.2 Poor Knowledge

Respondents were considered to have a “poor knowledge” of insulin self‐administration if they answered the knowledge assessment questions below the mean score.

#### 2.8.3 Good Practice

Respondents were considered to have a “good practice” of insulin self‐administration if they answered at or above the mean score of the knowledge assessment questions.

#### 2.8.4 Poor Practice

Respondents were considered to have a “poor practice” of insulin self‐administration if they answered the knowledge assessment questions below the mean score.

### 2.9 Ethical Approval and Consent to Participate

Ethical approval for the study and study protocol was obtained from the Wollo University College of Medicine and Health Sciences ethical review board (Ref: 0745/088/13/12). The aims of the study were clearly explained to the survey participants. The information was collected after obtaining written informed consent from each participant. The right was given to the study participants to refuse or discontinue participation at any time they wanted and the chance to ask anything about the study. For obscurity, the participant's name was not used at the time of data collection, all other personnel information was kept entirely obscure, and confidentiality was assured throughout the study period.

## 3 Results

### 3.1 A Sociodemographic‐Related Variable of Study Participant

In this study, 410 study participants were included, with a response rate of 97.4%, and 11 (2.6%) of the study participants did not volunteer to participate in the study. More than half of the participants were female 215 (52.4%), and the mean age of the study group was 41.3 (SD ± 16.2) years, with 99 (24.1%) of the participants being aged ≥ 55 years. In terms of education, 140 (34.1%) were illiterate, and 128 (31.2%) were housewives. Furthermore, 276 (67.3%) of the participants were married, and the family size of 283 (69%) participants was less than five children. About half of the study participants, 206 (50.2%), resided in urban areas (Table 1).

**Table 1:** Sociodemographic characteristics of study participants at government hospitals of Dessie town, Ethiopia, 2020.

Variable		Number	Percentage
Sex	Male	195	47.6
	Female	215	52.4
Age	18–24 years	73	17.8
	25–34 years	92	22.4
	35–44 years	52	12.7
	45–54 years	94	22.9
	≥ 55 years	99	24.1
Educational status	Unable to read and write	140	34.4
	Read and write only	34	8.3
	Primary education	70	17.1
	Secondary education	93	22.7
	Diploma and above	73	17.8
Occupation	Employed	62	15.1
	Merchant	80	19.5
	Farmer	70	17.1
	Housewife	128	31.2
	Unemployed	70	17.1
Marital status	Single	121	29.5
	Married	276	67.3
	Others*	13	3.2
Family size	< 5	283	69
	≥ 5	127	31
Residence	Urban	206	50.2
	Rural	204	49.8

* Widowed, divorced.

### 3.2 Clinical‐Related Characteristics of Study Participant

Regarding the family history of DM, only 62 (15.1%) of the study participants had a family history of DM and 141 (34.0%) of the study participants had been diagnosed with DM more than 9 years ago. Regarding the type of insulin treatment, 173 (42.2%) of the study participants had been taking insulin for 1–4 years, and 281 (68.5%) of them had T1DM. In addition, 353 (86.1%) had not developed chronic diabetic‐related complications, and hypertension was the most common comorbid condition in this study at 64 (15.6%). In this study, 354 (86.3%) of the study participants were not a member of the Ethiopian Diabetic Association (EDA) and 237 (57.8%) of the study participants were informed by healthcare professionals about DM and self‐administration of insulin (Table 2).

**Table 2:** Clinical characteristics of study participants at government hospitals of Dessie town, Ethiopia, 2020.

Variables		Number	Percentage
Family history of DM	Yes	62	15.1
	No	348	84.9
Diagnose of DM	< 1 years	37	9
	1–4 years	113	27.6
	5–9 years	119	29.0
	> 9 years	141	34.0
Mode of diagnosing	Accidentally	6	1.5
	By sign and symptom	404	98.5
Duration of Insulin therapy	< 1 years	63	15.4
	1–4 years	173	42.2
	5–9 years	113	27.6
	> 9 years	61	14.9
Types of DM	T1DM	281	68.5
	T2DM	129	31.5
Admitted to hospital with DKA or HHS or coma	Yes	110	26.8
	No	300	73.2
DM complications	No complications	353	86.1
	Sexual dysfunction	28	6.8
	Others*	29	7.1
Member of the Ethiopian Diabetic Association	Yes	56	13.7
	No	354	86.3
Dosing schedule	2 times a day	410	100
Comorbid illness	No comorbid illness	334	81.5
	Hypertension	64	15.6
	Others**	12	2.9
Means of information	Health profession only	237	57.8
	Mass media and health profession	119	29.0
	Others***	54	13.2

* Retinopathy, neuropathy, foot ulcer, retinopathy with neuropathy, retinopathy with sexual dysfunction, and neuropathy with foot ulcer.

** Liver disease, kidney problem, heart problem, kidney problem, and hypertension.

*** No information, relative, internet, media, health profession and relative, internet and book, health profession and books, health profession and books, and relative and internet.

### 3.3 Knowledge of Self‐Administration of Insulin

**Figure 1:**
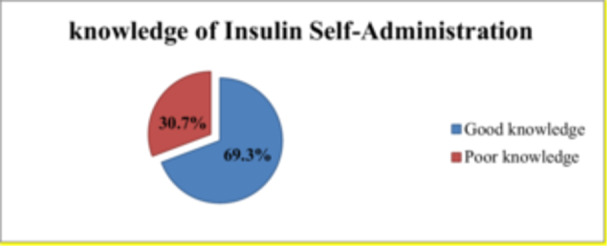
Insulin self‐administration knowledge in diabetic participants at government hospitals of Dessie town, Ethiopia, 2020.

According to the study, 69.3% (95% CI: 64.4%–73.7%) of participants had good knowledge of self‐administration of insulin (as shown in Figure 1). Out of the total participants, 209 (51.0%) had adequate knowledge of why insulin is prescribed for DM patients. Additionally, 214 (52.2%) of the participants knew that insulin controls blood glucose levels. Furthermore, 197 (48.0%) of the participants were aware that insulin injection cannot cure DM, and 191 (46.6%) knew the benefits of insulin injection. However, only 96 (23.4%) of participants were aware of the complications associated with insulin injection. When it comes to the storage of insulin, the study found that 373 (91.0%) participants were aware of the storage conditions (refrigerator or sand soaked with water). In terms of insulin administration practices, only 79 (19.3%) of the respondents were aware that post‐insulin injection massaging serves to diminish the rapid absorption of insulin. Additionally, only 89 (21.7%) of the participants knew that rotating the injection sites can reduce pain and prevent the wasting of subcutaneous tissue, and in Table 3, there is more detailed information on the study participants' knowledge of insulin self‐administration.

**Table 3:** Self‐administration of insulin knowledge in diabetic patients at government hospitals in Dessie town, Ethiopia, 2020.

Question		Number	Percentage
DM means high blood sugar level	Yes	294	71.7
	No	116	28.3
Do you know why insulin is prescribed for diabetic patients?	Yes	209	51
	No	201	49
Do you know that insulin cannot cure diabetics?	Yes	197	48
	No	213	52
Do you know that insulin controls blood glucose levels?	Yes	214	52.2
	No	196	47.8
Do you know the benefit of insulin in preventing the complications of DM?	Yes	191	46.6
	No	219	53.4
Do you know the complications of insulin injections?	Yes	96	23.4
	No	314	76.6
Do you know that insulin vial is stored in the refrigerator or cold place or sand soaked with water?	Yes	373	91
	No	37	9
Do you know that insulin injection is taken soon after or just before taking food?	Yes	289	70.5
	No	121	29.5
Do you know massage after injection is used to reduce the rapid absorption of insulin?	Yes	79	19.3
	No	331	80.7
Do you know the rotation of the injection site is to reduce pain and prevent wasting of subcutaneous tissue?	Yes	89	21.7
	No	321	78.3
Do you know the sites of insulin injections are the abdomen, thigh, glutei, and deltoid?	Yes	357	87.1
	No	53	12.9
Do you know insulin absorption is faster from the abdomen than other sites of injection?	Yes	94	22.9
	No	316	77.1
Do you know the benefits of insulin self‐administration are time‐saving, inexpensive, and easy to take on yourself while traveling?	Yes	345	84.1
	No	65	15.9
Do you know needles produced at home for insulin injection are infectious?	Yes	277	67.6
	No	133	32.4

### 3.4 Practice Toward Self‐Administration of Insulin

**Figure 2:**
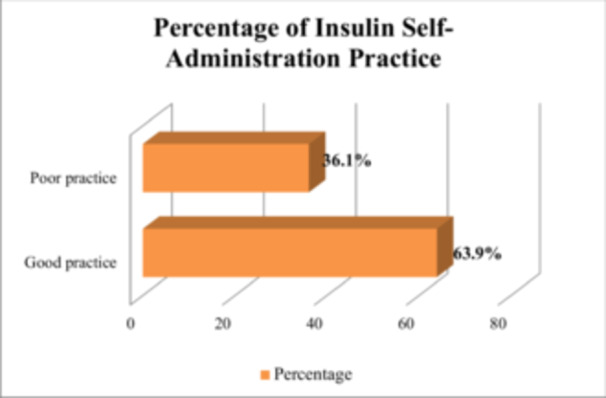
Practice toward self‐administration of insulin in diabetic participants at government hospitals of Dessie town, Ethiopia, 2020.

Out of the total of 410 participants in the study, 63.9% (with a 95% CI of 60%–69.3%) followed good practices related to insulin injection (as shown in Figure 2). Among the participants, 148 (35.4%) checked the expiry date of insulin, 266 (64.9%) changed the site of insulin injection frequently, 31 (7.6%) kept the insulin vial at room temperature for at least 15 min before injection, 65 (15.9%) removed air bubbles from the insulin syringe before injection, and 417 (99.3%) of the participants used a single syringe more than once. Further, 337 (82.7%) of the participants always carried an insulin injection tool when away from home, 339 (82.7%) performed a scheduled number of insulin injections, and 371 (90.5%) of the participants did not miss or skip insulin injections (as shown in Table 4).

**Table 4:** Practice of self‐administering insulin by diabetic patients at government hospitals in Dessie town, Ethiopia, in 2020.

Question	Answer	No	%
Do you check the expiration date of insulin?	Yes	148	36.1
	No	262	63.9
Do you change the site of injection frequently?	Yes	266	64.9
	No	144	35.1
Do you keep the insulin vial at room temperature for at least 15 min before injection?	Yes	31	7.6
	No	379	92.4
Do you wash your hands with soap and water before handling the injection devices?	Yes	138	33.7
	No	272	66.3
Do you remove the air bubbles from the insulin syringe before injecting?	Yes	65	15.9
	No	345	814.1
Do you use a single syringe for more than one time?	Yes	407	99.3
	No	3	0.7
Do you dispose of used insulin needles in a special container (household garbage bin) at home?	Yes	142	34.6
	No	268	65.4
Do you eat some food shortly after insulin injection?	Yes	328	80
	No	82	20
Do you clean and dry the site of injection before insulin administration?	Yes	133	32.4
	No	277	67.6
Do you always carry your insulin injection tool when away from your home?	Yes	337	82.2
	No	73	17.8
Each day, can you perform the scheduled number of insulin injections?	Yes	339	82.7
	No	71	17.3
Do you ever miss or skip an injection?	Yes	39	9.5
	No	371	90.5
Do you Inject your insulin when other people are around, without hesitation?	Yes	324	79
	No	86	21
Do you adjust the dose (units) of insulin when you are ill and have a fever, diarrhea, or lack of appetite?	Yes	98	23.9
	No	312	76.1

**Figure 3:**
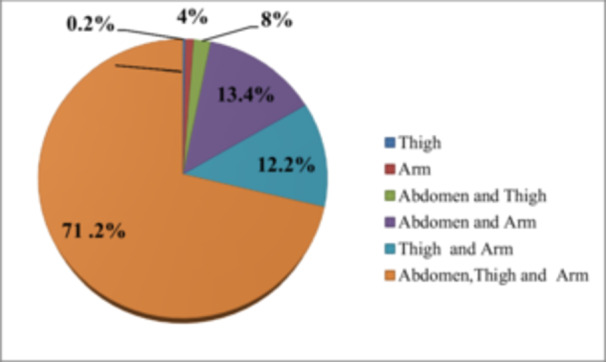
Insulin injection practice in diabetic participants at government hospitals of Dessie town, Ethiopia, 2020.

According to the assessment conducted on participants' insulin injection practices, 292 individuals (71.2%) were administering insulin on their abdomen, thigh, and arm, whereas 55 (13.4%) were injecting insulin on their abdomen and arm, and 50 (12.2%) were injecting on their arm and thigh (as shown in Figure 3).

**Figure 4:**
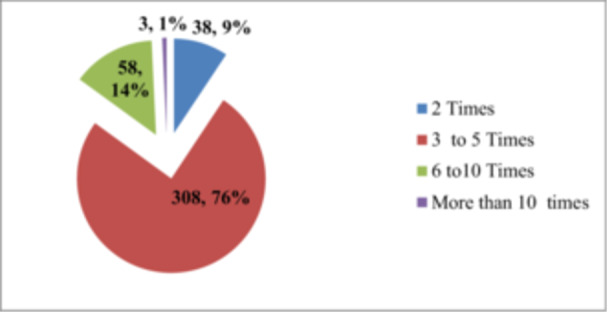
Single insulin syringe used to practice in diabetic participants at government hospitals of Dessie town, Ethiopia, 2020.

Of the total of 308 (75.1%) study participants, a single syringe was used 3–5 times. Of the respondents, 58 (14.1%) used a single syringe 6–10 times (Figure 4).

### 3.5 Factors Associated With Knowledge of Self‐Administration of Insulin

Univariate analysis revealed that 13 variables were associated with knowledge of self‐administration of insulin. Of these candidate variables, all were categorical variables, nine of which were multi‐categorical variables (age, education, occupation, marital status, family size, duration of diagnosis of diabetes, duration of insulin therapy, complication of DM, and Information about DM); the rest four were binary variables (residence, family history of diabetes, type of DM, and membership in the EDA). To further investigate the association between these variables, a multivariate binary regression analysis was conducted with 13 outcome variables. Finally, through cross‐validation using the hierarchical regression method, four variables were identified as significant for the good knowledge of self‐administration of insulin.

The occupation of an individual was found to be significantly associated with their knowledge of self‐administration of insulin. Being a merchant made a person 3.15 times more likely to have good knowledge (AOR, 3.15; 95% CI: 1.38–7.20, *p* = 0.007), while being employed made them 3.18 times more likely to have good knowledge (AOR, 3.18; 95% CI: 1.19–8.44, *p* = 0.02). Moreover, the type of diabetes was also found to be significantly associated with knowledge of self‐administration of insulin. People with type 2 diabetes were found to be 42% less likely to have good knowledge (AOR, 0.58; 95% CI: 0.35–0.963, *p* = 0.04). Membership in the EDA was also found to be significantly associated with knowledge of self‐administration of insulin. People without membership in the EDA were found to be 61% less likely to have good knowledge (AOR, 0.39; 95% CI: 0.17–0.92, *p* = 0.03). Lastly, information about DM was also significantly associated with knowledge of self‐administration of insulin. People who received information about DM from health professionals and mass media were found to be 2.12 times more likely to have good knowledge (AOR, 2.12; 95% CI: 1.08–4.14, *p* = 0.03) (Table 5).

**Table 5:** Factors associated with good knowledge toward self‐administration of insulin in diabetic participants at government hospitals of Dessie town, Ethiopia, 2020.

Variable	Categories	Knowledge of ISA		COR	AOR with CI	*p* value
		Good	poor			
Occupation	Farmer	38	32	1	1	
	Merchant	15	65	5.15 (2.47, 10.70)	3.15 (1.38, 7.20)	**0.007**
	Employed	11	51	5.51 (2.47, 12.29)	3.18 (1.19, 8.44)	**0.02**
	Housewife	48	80	1.98 (1.09, 3.57)	1.72 (0.94, 3.15)	**0.08**
	Unemployed	14	56	4.75 (2.24, 10.07)	3.39 (1.53, 7.52)	**0.003**
Types of DM	Type 1	77	204	1	1	
	Type 2	49	80	0.62 (0.39, 0.96)	0.58 (0.35, 0.96)	**0.04**
Member of EDA	Yes	8	48	1	1	
	No	118	236	0.33 (0.15, 0.73)	0.39 (0.17, 0.92)	**0.03**
Information about DM	Health professional only	96	141	1		
	Both health professionals and media	18	101	3.82 (2.17, 6.72)	2.12 (1.08, 4.14)	**0.03**
	Others	12	42	2.38 (1.19, 4.76)	1.19 (0.51, 2.80)	0.68

*Note:* Bold values indicate statistically significant at *p* < 0.05.

Abbreviations: AOR = adjusted odious ratio, CI = confidence interval, COR = crude odious ratio, ISA = insulin self‐administration.

### 3.6 Factors Associated With the Practice of Self‐Administration of Insulin

Univariate analysis revealed that 13 variables were associated with the practice knowledge toward self‐administration of insulin. To further investigate the association between these variables, a multivariate binary regression analysis was conducted with 13 outcome variables. Finally, through cross‐validation using the hierarchical regression method, only two variables were identified as significant with the good practice toward self‐administration of insulin.

The study found that age is a significant factor in the practice of insulin administration. Individuals aged 25–34 years were found to be 54.4% less likely to demonstrate good practice (AOR, 0.46; 95% CI: 0.22–0.94, *p* = 0.03). Similarly, those aged 35–44 years, 45–54 years, and 55 years or older were 69%, 67%, and 90% less likely to demonstrate good practice, respectively (AOR 0.306, 0.33, and 0.09, respectively, with corresponding *p* values of 0.02, 0.02, and < 0.001). Additionally, the duration of insulin therapy was found to be significantly associated with the practice of self‐administration of insulin. Those with a duration of insulin therapy of 5–9 years were 2.82 times more likely to demonstrate good practice (AOR, 2.82; 95% CI: 1.31–6.10, *p* = 0.008). Similarly, those with a duration of insulin therapy of more than 9 years were 4.54 times more likely to demonstrate good practice (AOR, 4.54; 95% CI: 1.58–13.11, *p* = 0.005) (Table 6).

**Table 6:** Factors associated with good practice of insulin self‐administration in diabetic participants at government hospitals of Dessie town, Ethiopia, 2020.

Variable	Categories	Practice of ISA		COR with CI	AOR with CI	*p* value
		Good	Poor			
Age	18–24 years	17	56	1	1	
	25–34 years	32	60	0.57 (0.28, 1.14)	0.46 (0.22, 0.94)	**0.03**
	35–44 years	16	36	0.68 (0.31, 1.52)	0.31 (0.11, 0.85)	**0.02**
	45–54 years	29	65	0.68 (0.34, 0.37)	0.33 (0.13, 0.82	**0.02**
	≥ 55 years	51	48	0.29 (0.15, 0.56)	0.09 (0.03, 0.31)	**0.001**
Duration of Insulin therapy	< 1 years	27	36	1	1	
	1–4 years	63	110	1.31 (0.73, 2.36)	1.69 (0.90, 3.19)	0.1
	5–9 years	36	77	1.60 (0.85, 3.03)	2.82 (1.31, 6.10)	**0.008**
	> 9 years	19	42	1.66 (0.79, 3.46)	4.54 (1.57, 13.11)	**0.005**

*Note:* Bold values indicate statistically significant at *p* < 0.05.

Abbreviations: AOR = adjusted odious ratio, CI = confidence interval, COR = crude odious ratio, ISA = insulin self‐administration.

## 4 Discussion

Insulin is commonly used in the management of both T1DM and T2DM. However, inadequate knowledge and malpractice on insulin self‐administration could result in poor treatment outcomes and insulin‐related complications like hypoglycemia. Therefore, the present study aims to assess the knowledge, practice, and associated factors of insulin self‐administration among patients with DM in Dessie Town Governmental Hospitals.

In this study, the proportion of good knowledge of insulin self‐administration was found to be 69.3% (95% CI: 64.4%–73.7%), which is in line with studies conducted in Ethiopia Zewuditu Memorial Hospital 63.4% [19], Bedele Hospital 67.3% [24], Alnamas 60% [25], and India (68%) [26]. However, the result was higher than the findings from Ethiopia in Oromia Metu 38.5% [8], Tigray Mekele 55.5% [9], Hawassa 56.1% [22], and Karad 52.5% [27]. This difference might be due to the methodology, sample size, sociodemographic characteristics of the studied subjects, differences in literacy level, access to optimal education, and demonstration of insulin self‐administration by healthcare providers.

The proportion of good practice in self‐administration of insulin was 63.9% (95% CI: 60%–69.3%). This study was higher than the study conducted in Iraq on the knowledge and practice of self‐administration of insulin 54% [28] and Nepal 52% [6]. It was also higher than another study conducted in Ethiopia Hawassa 54.4% [22]. The difference could also be due to the sociodemographic characteristics, management approach, or patient education in outpatient clinics regarding self‐administration of insulin, diagnosis of diabetes, and initiation of insulin therapy, which leads to differences in the proportion of practice regarding self‐administration of insulin compared to the other studies. It is important to know insulin complications when managing diabetes [29]. Poor self‐administration skills in insulin can lead to treatment‐related errors, resulting in the formation of lipodystrophies. These abnormalities can cause insulin malabsorption and reduce insulin effectiveness. To minimize the development of lipodystrophies and improve insulin absorption, it is important to educate diabetes patients on the importance of regularly rotating their injection sites. Recommended sites include the abdomen, thighs, upper arms, and buttocks [30, 31]. In this study, 314 (76.6%) had poor knowledge of insulin complications. To address this issue, proper instruction and demonstrations for self‐administering insulin injections are essential. Comprehensive education programs on insulin usage should empower individuals with DM and serve as vital components of effective diabetes management. A study conducted in Egypt found a statistically significant improvement in patient's knowledge, attitudes, and practices regarding the self‐administration of insulin injections among diabetic patients after a structured educational program compared to before the program [32].

This study found a correlation between occupation and knowledge. A merchant study participants were 3.47 times more likely to be knowledgeable (AOR, 3.15; 95% CI: 1.38–7.20) than farmers. Patients were 3.18 times (AOR, 3.18; 95% CI: 1.19–8.44) more likely to have good knowledge than farmers among employed study participants and 3.39 times (AOR, 3.39; 95% CI: 1.53–7.52) more likely to have good knowledge than farmers among unemployed study participants, which is consistent with other studies conducted in Nepal, and Alnam's occupation was significantly associated with good knowledge, respectively [6, 25]. The study found that participants with T2DM were 42% (AOR, 0.58; 95% CI: 0.35–0.96) less likely to have good knowledge compared to those with T1DM. This could be because patients with T1DM start insulin therapy earlier and continue it for a longer duration from the time of diagnosis, while patients with T2DM start taking insulin only after oral hypoglycemic agents fail. This means that those with T2DM began insulin injections for a shorter period, leading to less chance of receiving information and proper follow‐up education like those with T1DM, who acquire enough knowledge about self‐administration of insulin.

It was found that study participants who were not members of EDA were 61% (AOR, 0.39; 95% CI: 0.17–0.91) times less likely to have good knowledge about self‐administration of insulin as compared to those who were members of EDA. This may be because nonmembers of EDA lack access to additional diabetic‐related information, education, leaflets, and teaching aids, such as awareness creation services for self‐administration of insulin, which members of EDA have access to through the association's diabetic education, leaflets, books, and other awareness creation services. Participants who received information about DM from both health professionals and mass media were found to have a 2.12 times higher likelihood of good knowledge about DM (AOR, 2.12; 95% CI: 1.08, 4.14) compared to those who received information only from health professionals. This may be because mass media plays an important role in creating awareness in the community, which can enhance patients' knowledge of self‐administration of insulin.

The study found that only age and duration of insulin therapy are related to good insulin self‐administration practices. Patients aged 55 or above were 90% (AOR, 0.09; 95% CI: 0.03–0.31) less likely to have good practice compared to younger patients (aged 18–24). A similar study conducted in Bangalore [26] was less likely to have good practice compared to younger patients (aged 18–24). A similar study conducted in Bangalore also found that age was associated with good practices. This could be due to older patients having limited access to information about their disease and medication through the internet or leaflets and books. Additionally, self‐administration of insulin requires cognitive and psychomotor skills to learn different procedures, including storage, transportation, preparation, and application of the solution, as well as handling of syringes, needles, or injection pens. As age increases, cognitive and psychomotor skills may decrease, making it more challenging to practice correctly [15].

Participants in a study who had been on insulin therapy for a longer duration showed better insulin administration practice. Those who had been on insulin therapy for more than 9 years were 4.54 times (AOR, 4.54; 95% CI: 1.58–13.11) more likely to have good practice than those who had started insulin therapy less than a year ago. A similar study conducted in Bangalore also showed that the start of insulin therapy was associated with practice [26]. This may be because, with a longer duration of insulin therapy, patients have a higher chance of exposure to information and knowledge, which ultimately improves their self‐administration of insulin skills [32].

## 5 Limitations of the Study

The study had a cross‐sectional design, which means that it was not able to establish causal relationships. Additionally, the study was limited to a governmental referral hospital, so the results may not be representative of the knowledge and practices of patients with diabetes in other healthcare systems. The study aimed to evaluate the knowledge, practices, and factors related to insulin self‐administration in patients with DM, and the data was only collected from the Dessie Town Governmental Hospital. Therefore, the results cannot be generalized to DM patients in Ethiopia.

## 6 Conclusion

The study found that patients had suboptimal knowledge and practice regarding insulin self‐administration, particularly regarding the potential complications associated with insulin injection. It was also revealed that massaging after insulin injection can reduce the rapid absorption of insulin, and rotating injection sites can reduce pain and prevent the wasting of subcutaneous tissue. The study also determined that patients' occupation, type of diabetes, membership in EDA, and the source of information about diabetes (from health professionals or media) were associated factors that impacted their knowledge. Strategies should be implemented to address the specific needs of patients based on these factors. Therefore, comprehensive educational programs are essential for diabetes care, empowering patients to manage their condition effectively, bridging knowledge gaps, and enhancing insulin therapy outcomes. In addition, it is crucial to conduct a training program for diabetes patients on insulin therapy to enhance their knowledge and self‐administration skills. This program can be implemented in the outpatient clinic during follow‐up visits.

## Author Contributions

**Wasihun Melaku Feleke:** conceptualization, investigation, writing – original draft, methodology, formal analysis, project administration, data curation, writing – review and editing, visualization, validation, software, resources. **Caridad Sanchez Olis:** conceptualization, methodology, validation, writing – review and editing, project administration, supervision, software. **Adem Hussien Endris:** methodology, validation, visualization, writing – review and editing, formal analysis, data curation, supervision, software, conceptualization. **Seid Legesse Hassen:** conceptualization, investigation, methodology, validation, supervision, formal analysis, software, writing – review and editing. **Yimer Seid Ali:** conceptualization, writing – original draft, methodology, validation, visualization, writing – review and editing, formal analysis, software, supervision. **Dessale Abate Beyene:** conceptualization, writing – review and editing, visualization, validation, methodology, formal analysis, software, data curation, supervision, writing – original draft.

## Conflicts of Interest

The authors declare no conflicts of interest.

### 1 Transparency Statement

The WMF affirms that this manuscript is an honest, accurate, and transparent account of the study being reported; that no important aspects of the study have been omitted; and that any discrepancies from the study as planned have been explained.

## Data Availability

The data that support the findings of this study are available from the corresponding author upon reasonable request.
